# Influence of intrathecal delivery of bone marrow-derived mesenchymal stem cells on spinal inflammation and pain hypersensitivity in a rat model of peripheral nerve injury

**DOI:** 10.1186/s12974-014-0157-8

**Published:** 2014-09-12

**Authors:** Sabrina Schäfer, Julie V Berger, Ronald Deumens, Stéphanie Goursaud, Uwe-Karsten Hanisch, Emmanuel Hermans

**Affiliations:** Institute of Neuroscience (IoNS), Group of Neuropharmacology, Université catholique de Louvain, Avenue Hippocrate 54.10, 1200 Brussels, Belgium; EURON European Graduate School of Neuroscience, Maastricht, The Netherlands; Current address: Institute of Neurophysiology, Centre for Biomedicine and Medical Technology Mannheim, Medical Faculty Mannheim, Heidelberg University, Ludolf-Krehl-Str. 13-17, 68167 Mannheim, Germany; Institute of Neuropathology, University of Göttingen, Robert-Koch-Strasse 40, 37075 Göttingen, Germany

**Keywords:** Mesenchymal stem cells, Neuroinflammation, Microglia, PSNL, Pain

## Abstract

**Background:**

Multipotent mesenchymal stem (stromal) cells (MSCs) have been credited with immunomodulative properties, supporting beneficial outcomes when transplanted into a variety of disease models involving inflammation. Potential mechanisms include the secretion of paracrine factors and the establishment of a neurotrophic microenvironment. To test the hypothesis that MSCs release soluble mediators that can attenuate local inflammation, we here analysed the influence of MSCs on the activation of microglia cells, as well as on inflammatory parameters and pain behaviour in a surgical rat model of neuropathic pain.

**Methods:**

We focussed on an experimental model of partial sciatic nerve ligation (PSNL), characterised by a rapid and persistent inflammation in the dorsal lumbar spinal cord where sensory inputs from the sciatic nerve are processed. Via indwelling intrathecal catheters, MSCs were repetitively grafted into the intrathecal lumbar space. Animals were evaluated for mechanical and thermal hypersensitivity over a period of 21 days after PSNL. Afterwards, spinal cords were processed for immunohistochemical analysis of the microglial marker ionized calcium-binding adapter molecule 1 (Iba1) and quantification of inflammatory markers in ipsilateral dorsal horns. We hypothesised that injections on postsurgical days 2 to 4 would interfere with microglial activation, leading to a reduced production of pro-inflammatory cytokines and amelioration of pain behaviour.

**Results:**

PSNL-induced mechanical allodynia or heat hyperalgesia were not influenced by MSC transplantation, and spinal cord inflammatory processes remained largely unaffected. Indeed, the early microglial response to PSNL characterised by increased Iba1 expression in the lumbar dorsal horn was not significantly altered and cytokine levels in the spinal cord at 21 days after surgery were similar to those found in vehicle-injected animals. Grafted MSCs were detected close to the pia mater, but were absent within the spinal cord parenchyma.

**Conclusions:**

We conclude that intrathecal administration is not an appropriate route to deliver cells for treatment of acute spinal cord inflammation as it leads to entrapment of grafted cells within the pia mater. We propose that the early inflammatory response triggered by PSNL in the lumbar spinal cord failed to effectively recruit MSCs or was insufficient to disturb the tissue integrity so as to allow MSCs to penetrate the spinal cord parenchyma.

## Introduction

Partial sciatic nerve ligation (PSNL) is a commonly used animal model for studying mechanisms underlying neuropathic pain [1]. Hypersensitivity to mechanical and thermal stimuli (mechanical allodynia and thermal hyperalgesia) that develops rapidly after PSNL is at least partly dependent on synaptic sensitisation processes within the dorsal spinal cord to where sensory nerve endings project [2]. Indeed, at the cellular level, nerve injury leads to an activation of micro- and astroglial cells within the ipsilateral dorsal horn of the spinal cord concurrent with the release of a wide range of inflammatory mediators, including pro-inflammatory cytokines [3-5]. Glial cell activation has been implicated in the onset but also in the maintenance of pain hypersensitivity [6,7]. Given the lack of effective treatments for neuropathic pain [8], interventions targeting the neuroinflammatory activation cascades are considered as promising therapeutic perspectives. Indeed, several studies with different anti-inflammatory and glia-modulating drugs showed positive outcomes in a variety of experimental paradigms, some of which are currently being tested in clinical trials [3,9,10].

Convincing immunomodulatory features have been ascribed to mesenchymal stem (stromal) cells (MSCs) derived from the bone marrow, an easily accessible and highly proliferative stem cell source [11-14]. While the prominent benefit provided by these multipotent adult stem cells has been largely documented in several disease models, being characterised by inflammatory reactions in the nervous system (such as amyotrophic lateral sclerosis (ALS), Parkinson’s disease, Alzheimer’s disease or traumatic injuries), it is noteworthy that the immuno-modulatory capacity of grafted MSCs does not necessarily depend on cell-specific differentiation or the integration of the grafted cells into the host tissue. It rather seems that MSCs possess the potential to establish a transient neurotrophic microenvironment that is beneficial and supports tissue healing, repair and regeneration. In fact, grafted MSCs have been recently described as “*in vivo* drugstores”, synthesising and secreting paracrine factors which mediate therapeutic benefits [15-18]. Supporting this concept, two *in vitro* studies have documented that MSCs can release growth/neurotrophic factors as well as anti-inflammatory proteins and modulate microglial responses to pro-inflammatory stimuli [19,20]. Moreover, single intra-brain or intravenous injections were shown to ameliorate neuroinflammation and associated behaviour in animal models of neuropathic pain, such as sciatic nerve constriction, contusion injury or spared nerve injury [21-23].

In the present investigation, we validated the immunomodulatory effect of MSCs on primary microglial cultures activated by bacterial lipopolysaccharide (LPS), a prototypical agonist of Toll-like receptor (TLR)4 serving as a standard stimulus to trigger pro-inflammatory microglial reactions. *In vivo*, MSCs were then repetitively injected via the intrathecal route between 2 and 4 days after PSNL or sham surgery. In this specific experimental set up, behavioural assessments were performed to explore whether the putative anti-inflammatory effect of MSCs would influence PSNL-evoked mechanical allodynia and thermal hyperalgesia.

## Material and methods

### Materials for cell culture

Culture media, foetal bovine serum (FBS), horse serum (HS), penicillin-streptomycin, fungizone, proline, trypsin-EDTA and PCR primers were purchased from Invitrogen (Merelbeke, Belgium). Poly-L-lysine was obtained from Sigma (Bornem, Belgium) and ultrapure *Escherichia coli* LPS from InvivoGen (Toulouse, France). Percoll™/RediGrad™ reagent was acquired from GE Healthcare (Uppsala, Sweden). TriPure RNA Isolation Reagent was bought from Roche Diagnostic (Vilvoorde, Belgium) and iScript cDNA Synthesis Kit as well as IQ™ SYBR® Green supermix from BioRad Laboratories (Nazareth, Belgium). Culture plastic ware was purchased from Greiner Bio-one (Wemmel, Belgium).

### Generation of rat bone marrow-derived mesenchymal stem cells and collection of conditioned medium

All experiments were performed in strict accordance with the European Union directive of 22 September 2010 (2010/63/EU). For all *in vivo* experiments, female Sprague Dawley rats were used and obtained from Charles River (Brussels, Belgium). MSCs were isolated from the bone marrow of tibias and femurs of 8-week-old Sprague Dawley rats and cultured in uncoated tissue culture flasks, as these cells readily adhere to the plastic. Cells were propagated for 15 passages and then characterised as previously described [24,25]. MSCs were harvested by trypsinisation and plated at a density of 1 × 10^5^ cells per well in six-well plates in 2 ml DMEM with high glucose, 10% FBS, 1% penicillin-streptomycin and 1% fungizone. For *in vitro* experiments, the medium was changed after 24 hours and cells were incubated for another 48 hours. Supernatant (MSC-conditioned medium) was then collected and centrifuged at 174 × *g* for 5 minutes to remove cell debris. Control medium was incubated in culture flasks (containing no cells) for 48 hours and also collected and centrifuged. MSC-conditioned and control media were frozen and stored at -20°C until further use. It is worth mentioning that the *in vitro* part of this study only examined the influence of conditioned medium from MSCs on cultured microglia and, therefore, the number of MSCs used to generate this conditioned medium cannot be compared to the number of cells used in the *in vivo* protocol where the cells were directly injected intrathecally.

### Primary culture of microglial cells

Primary cultures were obtained from the cerebral cortices of newborn wild-type Wistar rats as described before [26]. In brief, cells were obtained by a sedimentation protocol and plated into 75 cm^2^ poly-L-lysine-coated flasks. The culture medium was changed every 5 days. After 14 days of culturing, cells were trypsinised and microglia were separated from other cell types using a Percoll-density-gradient centrifugation. The resulting cultures were highly enriched in microglial cells and plated at a density of 3 × 10^4^ cells/cm^2^ in the same medium as used for MSC cultures.

### Activation of microglial cells

Twenty-four hours after plating, cell treatments were initiated by either incubating cells for 24 hours in 2 ml MSC-conditioned medium or in 2 ml control medium. Then the media were removed and replaced by fresh MSC-conditioned medium or control medium, this time with or without LPS (100 ng/ml), leading to four different culture conditions. After a 24-hour incubation period, the cell plates were transferred onto ice, washed with 1 × PBS, and TriPure Isolation Reagent was added to each well. Until RNA extraction, samples were stored at -20°C. This experiment was conducted in duplicates and repeated six times.

### RNA extraction, reverse transcription and real-time PCR

RNA was extracted according to the manufacturer’s protocol and treated with DNAse at 37°C for 30 minutes. Reverse transcription was carried out with the iScript cDNA Synthesis Kit, using 1 μg RNA in a total reaction volume of 20 μl. Real-time quantitative PCR was performed for quantification of the genetic expression of TNFα and IL-1β using the iCycler real-time PCR detection system (BioRad). Each reaction was carried out in a total volume of 25 μl, containing 2 ng cDNA template, 350 nM of both specific forward and reverse primers (TNFα forward: 5′-CCCCGACTATGTGCTCCTCAC-3′, TNFα reverse: 5′-AGGGCTCTTGATGGCGGA-3′; IL-1β forward: 5′-GGAAGGCAGTGTCACTCATTGTG-3′, IL-1β reverse: 5′-GGTCCTCATCCT GGAAGCTCC-3′) and the IQ SYBR Green super mix. The protocol consisted of 50 amplification cycles, each conducted as follows: 15 seconds of denaturation at 95°C, 45 seconds annealing at 60°C and 15 seconds of elongation at 79°C. For quantitative analysis, a standard curve was generated by amplification of serial dilutions of a cDNA template mix, consisting of all analysed samples. Each sample was normalised to the relative amplification of the housekeeping gene glyceraldehyde 3-phosphate dehydrogenase (GAPDH). Raw data was analysed by the “post-run data analysis” software provided by BioRad.

### Behavioural testing

For behavioural analyses, animals were first habituated to the testing procedures and surroundings. Procedures to examine hyperalgesia and allodynia have been previously described by our group in more detail [2]. To evaluate thermal sensitivity, a Paw Thermal Stimulator (San Diego University, CA, USA) was used, applying a beam of radiant heat to the mid plantar surface of rat’s hind paws (Hargreaves test). Paw withdrawal latencies (PWL) were measured for both hind paws with a cut-off set at 20 seconds to avoid tissue damage. For each time point, the average PWL of at least four testing trials, each separated by a 10-minute interval, was calculated per rat and normalised to the baseline value (PWL before surgery). Mechanical sensitivity was assessed with nine different von Frey filaments (Stoelting Co., Wood Dale, IL, USA) with a bending force from 0.4 g to 26 g, to determine the 50% paw withdrawal threshold (PWT), as previously described [27]. Results are shown as a percentage of the baseline value for each animal. The baseline values for the rats were obtained by taking the average PWL and PWT obtained in the 3 days preceding surgery. To rule out that nociceptive thresholds were influenced by the catheter implantation, PWL and PWT before and after catheter implant were compared. Postoperative assessments of pain behaviour were performed for 3 weeks after surgery, more specifically on days 1, 2, 3, 4, 5, 6, 7, 10, 14, 17 and 20. With respect to the behavioural examinations, a total of 9 to 11 animals were included in each group.

### Intrathecal catheterisation

Previous studies proposed that the female system mediating pathological pain has a substantially higher clinical relevance than that of males, which essentially relies on a TLR4-testosterone dependent pathway [28]. Also considering the higher incidence of chronic pain in women, only female rats were used in the present study. Three-month-old animals were anaesthetised by combined intraperitoneal injection of xylazine (10 mg/kg) and ketamine (80 mg/kg) and intrathecal polyethylene catheters were implanted by first incising the atlanto-occipital membrane and underlying dura mater and then carefully advancing the tip of the catheter along the spinal cord for a distance up to 8.5 cm, thereby reaching the lumbosacral enlargement. Skin layers were closed with 2-0 sutures. After surgery, bladders were manually voided and animals received 5 ml subcutaneous saline solution for rehydration. As long as animals were anaesthetised, eyes were regularly moistened with drops of isotonic NaCl solution. Topical antibiotic treatment of the sutured wound with Aureomycin® (Chlortetracyclin 1%, Erfa Pharm, Brussels, Belgium) took place right after surgery and once a day for the next 2 days. To keep catheters free and unblocked by cell-clots, they were flushed daily with 15 μl NaCl solution until cell treatment started. Animals showing neurological deficits after surgery were immediately euthanised. Several days after surgery, 15 μl 1% Xylocaine® (Lidocaine-hydrochloride 10 mg/ml, AstraZeneca, Brussels, Belgium) was injected intrathecally to verify that the catheter was positioned correctly, causing temporary deficits in hind limb function.

### Partial sciatic nerve ligation

Nine days after catheterisation, the animals were anaesthetised with sevoflurane (8% for induction; 4% for maintenance; oxygen as carrier gas) and PSNL was performed as previously described [2]. In brief, the right sciatic nerve was exposed at the mid-thigh level and isolated from surrounding tissues. With a 6-0 suture, one-third to one-half of the nerve trunk was tightly ligated above its bifurcation in peroneal and tibial branches. Muscle and skin layers were finally closed with a 2-0 suture. Sham surgeries involved identical procedures, but without ligation of the sciatic nerve.

### Stem cell injection

**Figure 1:**
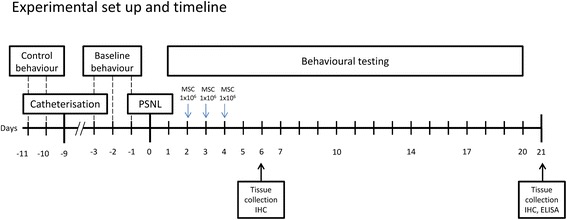
**Experimental design for** ***in vivo*** **experiments.** ELISA, enzyme-linked immunosorbent assay; IHC, immunohistochemistry; MSC, mesenchymal stem (stromal) cell; PSNL, partial sciatic nerve ligation.

Considering our previous studies with MSCs maintained in cultures for a prolonged time [24], we here exclusively used cells grown *in vitro* for 15 passages, which correspond to approximately 4 to 5 months. MSCs were first labelled *in vitro* by adding bromodeoxyuridine (BrdU, Sigma) (10 μM) to the culture medium 72 hours before cell harvesting. Suspensions of MSCs were then obtained by trypsinisation, two washes in PBS and resuspension in OptiMEM (Invitrogen) at a density of 66,000 cells/μl (1 million in 15 μl) for injection. This protocol was selected as it permits the delivery of a large amount of cells in a minimal volume, without clustering cells clotting the catheters, which we observed during preliminary tests using 2 million cells in 15 μl. Fifteen μl of MSC suspension or vehicle (OptiMEM) were then slowly injected intrathecally followed by a 10 μl saline flush. Avoiding any interference with the daily behavioural measurements, cell injections took place in the evenings. Cells were administered on days 2, 3 and 4 following surgery, as summarised in Figure 1. Leftovers of cells prepared for treatment were re-cultured to control viability and BrdU-positivity after a few cell cycles.

### Tissue collection

For immunohistochemistry, rats were killed by transcardiac perfusion with warm (37°C) saline solution (0.9% NaCl) followed by cold 4% paraformaldehyde (dissolved in PBS) under deep anaesthesia with xylazine (10 mg/kg) and ketamine (80 mg/kg). Immediately after perfusion, the spinal cord was isolated via hydro-extrusion and post-fixed overnight in 4% paraformaldehyde at 4°C. Tissues were cryo-protected by incubation in 15% sucrose solution (in PBS) for 24 hours at 4°C, followed by incubation in 30% sucrose solution for 2 days at 4°C. The lumbar region of each spinal cord (L4-L6) was quickly frozen (3 minutes in isopentane at -80°C) and stored at -80°C until cryosectioning. In total, 3 to 4 spinal cord samples were collected per group on day 6 post-surgery and 5 to 7 samples per group on day 21 post-surgery. They were serially cut at -30°C to obtain 10 series of transverse 25 μm thick sections, which were collected on ThermoScientific superfrost plus glass slides (VWR International, Leuven, Belgium). Sections were stored at -20°C until histological staining.

For ELISA, rats were euthanised with CO_2_ and decapitated. The spinal cord was rapidly isolated by hydro-extrusion and transferred in cooled PBS solution to carefully remove meninges. The lumbar enlargement was isolated and ipsilateral and contralateral sides were separated using the ventral fissure as a reference. Subsequently, these halves were divided into dorsal and ventral parts, using the central canal as a reference. Tissues were stored in microtubes at -80°C until used for analysis. For this experiment, 4 to 6 animals were used per group.

### Immunohistochemistry and quantification

After thawing and drying overnight at 37°C, every tenth section was stained for ionized calcium-binding adapter molecule 1 (Iba1) and BrdU. For Iba1 staining, sections were washed three times (3 × 10 minutes) in PBS before incubation in a blocking solution (5% HS, 1% Triton in PBS) for 90 minutes at room temperature (RT). The primary antibody against Iba1 (rabbit anti-rat Iba1, 1:1,000, Wako Pure Chemical Ltd, Osaka, Japan) was diluted in a PBS working solution containing 1% HS and 1% Triton for an overnight incubation at 4°C. The next day, after three washing steps (3 × 10 minutes) with PBS, sections were incubated with the secondary antibody (donkey anti-rabbit-Alexa488 antibody, 1:500, Invitrogen) diluted in the same working solution for 1 hour at RT. After three washes in PBS, nuclei were stained with DAPI (1:5,000) for 15 minutes at RT and, after washing sections three more times, they were finally embedded in Fluoprep and cover-slipped.

For BrdU detection, DNA was denatured by boiling the sections for 20 minutes in 10 mM citric acid at 96-98°C. Afterwards, sections were allowed to cool down (within the citric acid) for 30 minutes before being washed three times (3 × 10 minutes) in PBS with 0.5% Triton. After blocking for 90 minutes with 15% HS and 0.5% Triton in PBS at RT, sections were incubated with the primary antibody diluted in blocking solution (mouse anti-BrdU, 1:200, AbD Serotec, Düsseldorf, Germany) for 2 nights at 4°C. After three washes in PBS with 0.5% Triton, sections were incubated with the secondary antibody (goat anti-mouse-Alexa555 antibody, 1:500, Invitrogen) diluted in PBS with 0.5% Triton for 1 hour at RT. After three washes in PBS, nuclei were stained with DAPI (1:5,000) for 15 minutes at RT; sections were washed three more times and finally embedded in Fluoprep followed by cover-slipping.

Photo-micrographs of the ipsilateral dorsal horns were taken using a fluorescent inverted microscope (EVOS *fl*, AMG, Westburg, Leusden, The Netherlands) with a 4× objective. An average of 7 sections (L4-L5) per animal were analysed using ImageJ 1.44. The dorsal horn was delineated and, after background subtraction, Iba1-stained dorsal horns were analysed. The mean staining intensity in the dorsal horn was calculated for each photo-micrograph and data were plotted.

### ELISA

Tissue samples were suspended in 200 μl lysis buffer (Cell Signaling via New England Biolabs, Frankfurt, Germany) and 2 μl PMSF (200 mM), then incubated on ice for 5 minutes, sonicated and cleared by centrifugation (14,000 × *g*, 4°C, 10 minutes). Supernatants were collected and their protein content was determined with a MicroBCA assay (Pierce, Thermo Scientific, Bonn, Germany). IL-1β, IL-6, IL-10 and TNFα were measured using respective ELISA systems (R&D Systems, Wiesbaden, Germany) according to the manufacturer’s instructions. Absorbance was measured at 450 nm (with 540 nm reference) using an iMark microplate reader (Bio-Rad, Munich, Germany).

### Statistical analyses

All data are presented as means ± SEM and statistical analyses were performed with GraphPad Prism version 5.02 (GraphPad software, San Diego, CA, USA). Groups were compared by a two-way analysis of variance, followed by a Tukey *post-hoc* test correcting for multiple comparisons. To determine differences between the curves of behavioural data, the area under the curve was calculated for each animal and averaged per treatment group, so that treatment groups could be compared by a two-tailed student’s *t*-test. Day-by-day group differences between sham and PSNL or treated and untreated, respectively, were calculated via whole sample *t-*tests. Significant differences are indicated if *P* values were below 0.05.

## Results

### Mesenchymal stem cell-conditioned medium reduces microglial cytokine expression in response to lipopolysaccharide

**Figure 2:**
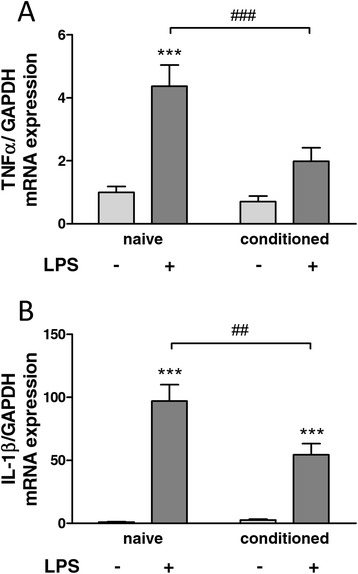
**Modulation of lipopolysaccharide-induced expression of inflammatory cytokines in primary microglial cultures.** The relative mRNA expression (normalised to glyceraldehyde 3-phosphate dehydrogenase; GAPDH) of the pro-inflammatory cytokines TNFα **(A)** and IL-1β **(B)** was examined in microglial cultures incubated for 48 hours in medium conditioned or not with MSCs and with or without lipopolysaccharide (LPS; 100 ng/ml) during the last 24 hours. Data shown are mean values with SEM from six independent experiments, analysed by two-way analysis of variance followed by Tukey *post-hoc* test. ^##^ *P* < 0.01 and ***/^###^ *P* < 0.001. Symbols denote significant differences between LPS-activated and non-activated groups (*) and between control and conditioned medium-treated groups (^#^).

*In vivo*, neuroinflammation involves activation of microglia, characterised by morphological changes, cell proliferation and production of pro-inflammatory cytokines. To test our hypothesis that MSCs release soluble mediators which can attenuate inflammation after peripheral nerve injury (PNI), we first evaluated the influence of MSC-conditioned medium on primary microglial cultures exposed to an inflammatory stimulus (LPS). We quantified the mRNA expression of the two key pro-inflammatory cytokines TNFα and IL-1β (Figure 2). As expected, exposure of microglial cells to LPS for 24 hours increased the gene expression of both targets. This increased expression induced by LPS could be significantly attenuated in cells that had been exposed to MSC-conditioned medium beforehand, demonstrating the immunomodulatory properties of soluble factors released by MSCs.

### Pain hypersensitivity is unaffected by mesenchymal stem cell transplantation

**Figure 3:**
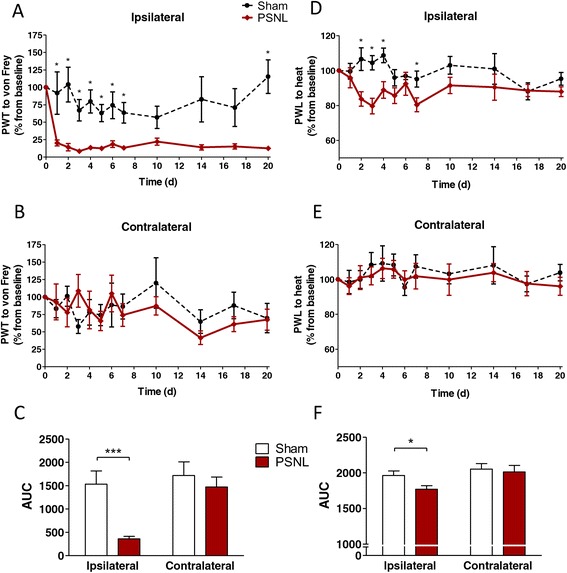
**Mechanical and thermal hypersensitivity after partial sciatic nerve ligation.** Rats were tested for mechanical hypersensitivity **(A-C)** by using von Frey filaments, and for thermal hypersensitivity **(D-E)** with the thermal paw stimulator during 20 days after partial sciatic nerve ligation (PSNL) surgery. Paw withdrawal threshold (PWT) of the ipsilateral **(A)** or contralateral **(B)** hindpaws after mechanical stimulation are expressed as percentage of the baseline PWT. Paw withdrawal latencies (PWL) of the ipsilateral **(D)** or contralateral **(E)** hindpaws after thermal stimulation are expressed as percentage of the baseline PWL. **(C,F)** Area under the curve (AUC) analysis of the corresponding graphs. Data shown are mean values with SEM. For AUC: *P < 0.05, *** *P* < 0.001, analysed by two-tailed student’s *t*-test. Day-by-day differences between groups were calculated by whole sample *t-*tests with * at least *P* < 0.05. Each group consisted of 9 to 11 animals.

Mechanical and thermal sensitivity were assessed during 20 days after sham or PSNL surgery. Figure 1 summarises the different steps and time points of the experimental design. When ipsilateral hind paws were stimulated with von Frey filaments, a clear and significant decrease in the PWT could be observed in PSNL animals when compared to their respective sham group. In contrast, no change in PWT was observed upon stimulation of the contralateral hind paws (Figure 3A-C). Heat stimulation of hind limbs led to a very similar outcome, although the decrease in the PWL was less prominent (Figure 3D-F). Altogether, these results indicate that hypersensitivity after PSNL is efficiently validated by the two behavioural tests.

**Figure 4:**
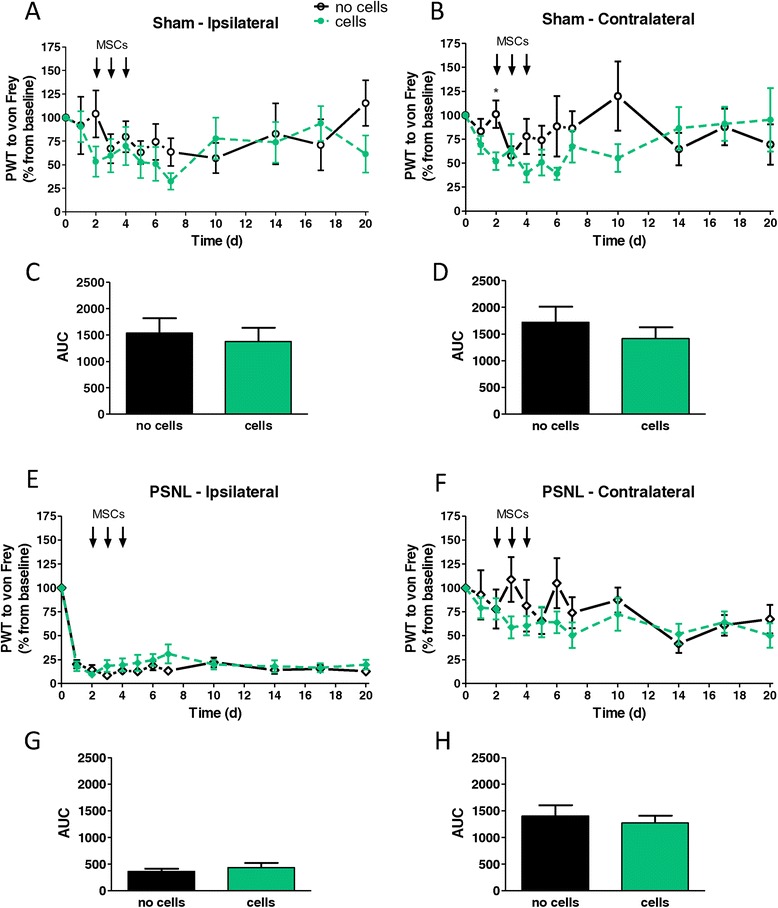
**Effect of intrathecally injected mesenchymal stem cells on mechanical hypersensitivity after partial sciatic nerve ligation.** Sham- **(A,B)** or partial sciatic nerve ligation (PSNL)- **(E,F)** operated animals either injected with vehicle (“no cells”) or mesenchymal stem cells (MSCs; “cells”) were tested by using von Frey filaments for 20 days after surgery. Paw withdrawal thresholds (PWT) of the ipsilateral (**A** or **E**) or contralateral (**B** or **F**) hindpaws are expressed as percentage of the baseline PWT. **(C,D** and **G,H)** Area under the curve (AUC) analysis of the corresponding graphs, analysed by two-tailed student’s *t*-test; data shown are mean values with SEM. Day-by-day differences between groups were calculated by whole sample *t*-tests with * at least *P* < 0.05. Each group consisted of 9 to 11 animals.

**Figure 5:**
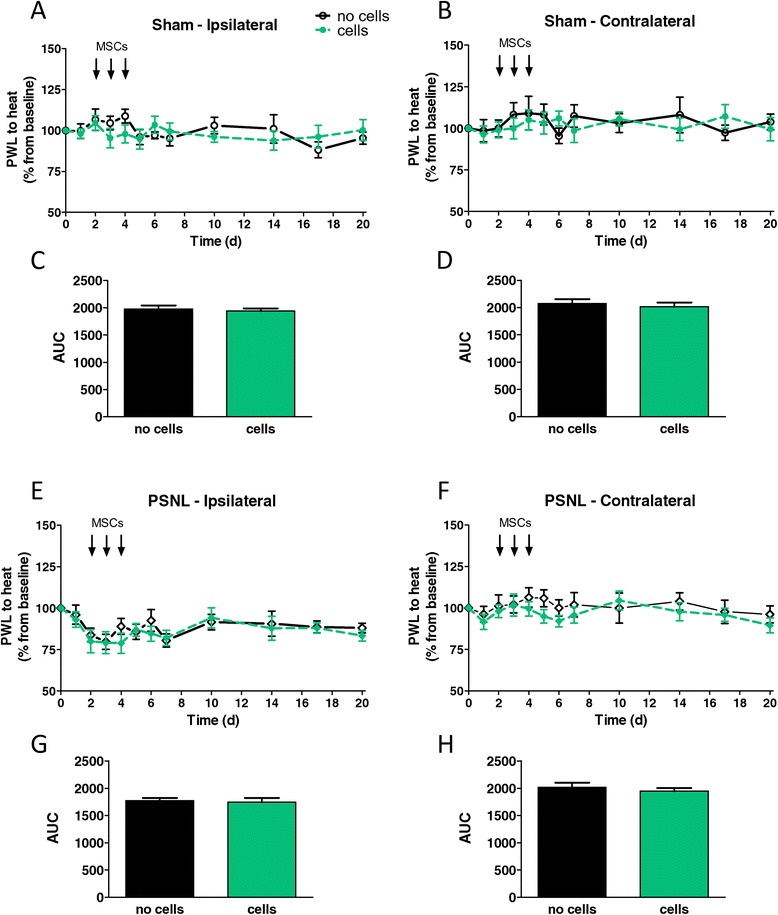
**Effect of intrathecally injected mesenchymal stem cells on thermal hypersensitivity after partial sciatic nerve ligation.** Sham- **(A,B)** or partial sciatic nerve ligation (PSNL)- **(E,F)** operated animals either injected with vehicle (“no cells”) or mesenchymal stem cells (MSCs; “cells”) were tested with the thermal paw stimulator for 20 days after surgery. Paw withdrawal latencies (PWL) of the ipsilateral (**A** or **E**) or contralateral (**B** or **F**) hindpaws are expressed as percentage of the baseline PWL. **(C,D** and **G,H)** Area under the curve (AUC) analysis of the corresponding graphs, analysed by two-tailed student’s *t*-test; data shown are mean values with SEM. Each group consisted of 9 to 11 animals.

Animals received vehicle or MSC injections in the evenings of day 2, 3 and 4 after surgery, when peripheral nerve injury-induced microglial activation was clearly established [29]. Analysis of mechanical hypersensitivity in sham-operated animals did not reveal any influence of MSC transplantation (Figure 4A-D). MSC injection did also not affect established PSNL-induced mechanical hypersensitivity (Figure 4E-H). In accordance with these data, thermal hypersensitivity was not influenced by MSC transplantation, neither after PSNL nor after sham surgery (Figure 5).

### Iba1 protein expression is increased after partial sciatic nerve ligation but not significantly affected by mesenchymal stem cell injection

**Figure 6:**
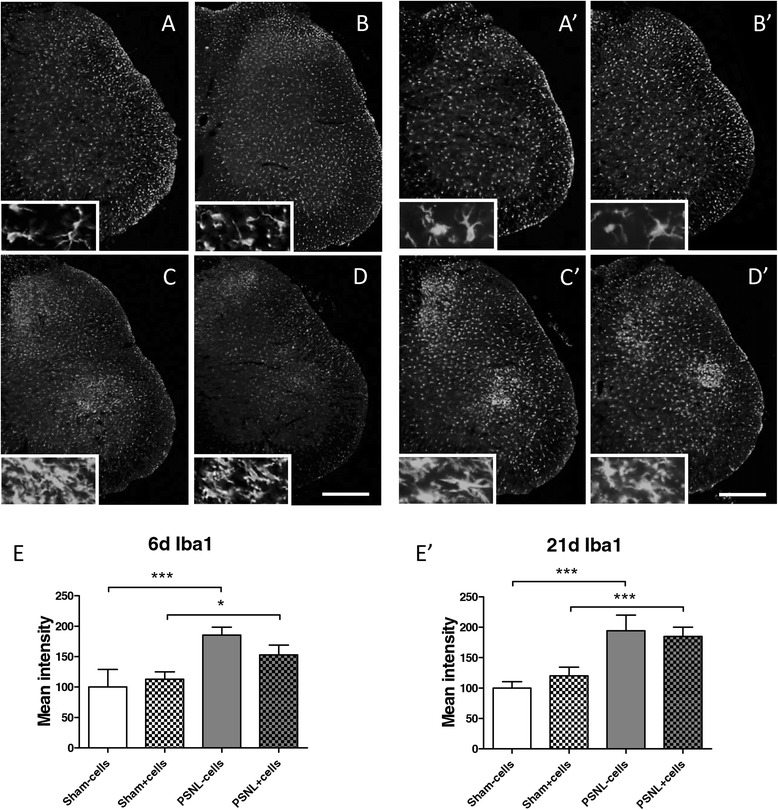
**Ionized calcium-binding adapter molecule 1 protein expression 6 days and 21 days after partial sciatic nerve ligation.** Immunohistological staining and quantification of the microglial marker ionized calcium-binding adapter molecule 1 (Iba1) at 6 days **(A**-**E)** and at 21 days **(A’**-**E’)** on the ipsilateral side of the lumbar spinal cord with low and high magnifications. Iba1 staining of sham-operated animals either vehicle-injected **(A,A’)** or MSC-injected **(B,B’)** and PSNL animals with vehicle **(C,C’)** or MSCs **(D,D’)**. Scale bar represents 500 μm. **(E,E’)** Quantification of the mean grey intensity within the total dorsal horn with SEM, two-way analysis of variance and Tukey *post-hoc* test for statistical analysis, *P < 0.05 and **P < 0.01. Groups at 6 days after PSNL consisted of 3 to 4 animals and groups at 21 days after PSNL consisted of 5 to 7 animals.

Immunohistochemical analysis of Iba1 protein expression was performed on cryosections of the lumbar spinal cord (L4-L5) at two different time points: 6 and 21 days after surgery. For MSC-treated animals, we hypothesised that microglial activation would trigger the recruitment of the injected MSCs and/or that MSCs would induce subsequent mitigation of microglial activation. In sham-operated animals, microglia were characterised as small and ramified cells (Figure 6A), while PSNL induced hypertrophied microglia with less extensive cellular ramification, leading to a prominent increase in Iba1 immunostaining in the ipsilateral dorsal horn (Figure 6C). MSC injections did not change the Iba1 immunostaining in sham-operated animals (Figure 6B) but they did seem to have an effect in PSNL animals, as the immunostaining appeared weaker and less diffuse in the dorsal horn, compared to the tissues from vehicle-treated PSNL animals (Figure 6C,D). Quantification of the mean staining intensity confirmed a significant increase after PSNL (Figure 6E), which was less pronounced after MSC injection, even though this difference failed to be statistically significant. At the end of the study, sham animals obviously still presented a “non-activated” morphology of microglia, which was not influenced by MSC injection (Figure 6A’,B’). PSNL animals still showed a robust Iba1 immunoreactivity in the dorsal horn with strongly hypertrophied microglia. These characteristics of microglial activation were not influenced by MSC injections in the early post-injury period, as indicated in Figure 6C’,D’. Thus, quantitative analysis of the mean Iba1 staining intensity showed an 94% increase for vehicle-treated PSNL animals compared to 85% for MSC-treated PSNL animals (no statistically significant difference) (Figure 6E’).

### Transplanted mesenchymal stem cells do not integrate into the spinal cord tissue

**Figure 7:**
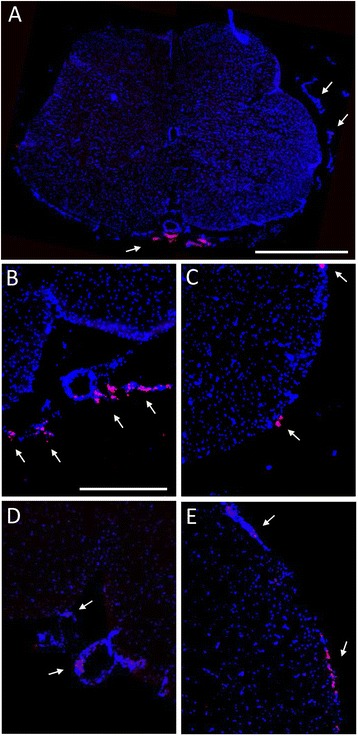
**Fate of mesenchymal stem cells after intrathecal injection 6 days and 21 days post-partial sciatic nerve ligation.** Immunohistological staining of the lumbar spinal cord (L4/L5) of partial sciatic nerve ligation (PSNL) animals injected with mesenchymal stem cells (MSCs). Bromodeoxyuridine (BrdU)-labelled MSCs appear red and cell nuclei blue. Cells were only validated as BrdU-positive when also showing a blue labelling. **(A)** Full spinal cord section 6 days after PSNL. Scale bar represents 1000 μm. **(B,C)** Representative photographs 6 days after PSNL. Scale bar represents 400 μm. **(D,E)** Representative photographs 21 days after PSNL. Arrows indicate BrdU-positive cells.

Prior to injection, MSCs were cultured in the presence of the thymidine analogue BrdU to facilitate their detection in the white and grey matter of the spinal cord and to investigate their differentiation potential after grafting into the tissue. However, as shown in Figure 7, MSCs did not integrate into the grey matter. A few BrdU-positive cells were found in sections from animals sacrificed 6 days after surgery (2 days after the last injection), and were located mainly around the spinal cord parenchyma in the leftovers of the pia mater spinalis. In most cases, grafted cells were found in the area of the anterior spinal artery (Figure 7A,B). Twenty-one days after surgery (17 days after the last injection), MSCs could still be detected, but the number of cells appeared to be lower than after 6 days (Figure 7D,E). The location appeared to be very similar as compared to the earlier time point, with the majority of cells being trapped in the pia mater spinalis. In a few animals, a few isolated BrdU-positive cells could be detected in the white matter, but this observation was not representative for all animals.

### Mesenchymal stem cells lack a long-term paracrine influence on cytokine release

**Figure 8:**
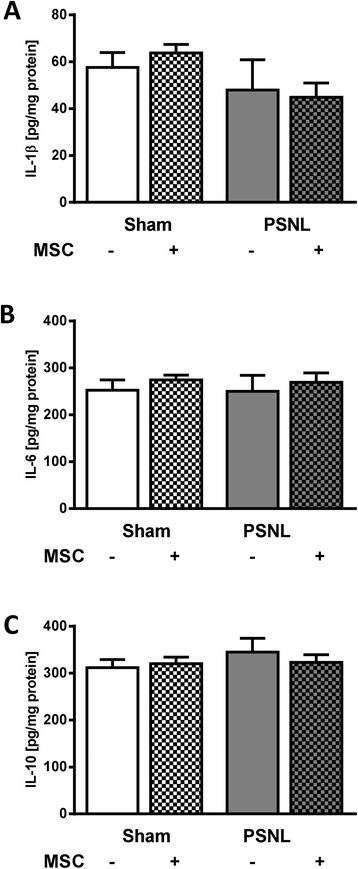
**Cytokine production in the ipsilateral dorsal horns 21 days post-partial sciatic nerve ligation.** Concentrations of **(A)** IL-1β, **(B)** IL-6 and **(C)** IL-10 were measured in supernatants of lysed tissues as derived from the ipsilateral dorsal horns of the L3-L6 spinal cord region. Concentrations of TNFα were below the detection level. Data represent mean with SEM, analysed by two-way analysis of variance followed by Tukey *post-hoc* test. Each group consisted of 4 to 6 animals. MSC, mesenchymal stem cells; PSNL, partial sciatic nerve ligation.

The concentration of the cytokines TNFα, IL-1β, IL-6 and IL-10 in lumbar spinal cord tissue from the ipsilateral dorsal horns (21 days after PSNL) was analysed and quantified by ELISA. The concentration of TNFα was below the detection level in all samples. Despite the significant increase in microgliosis 3 weeks after PSNL, we could not show any influence of the ligation on cytokine concentrations. As indicated in Figure 8, intrathecal injection of MSCs did not influence the spinal levels of IL-1β, IL-6 and IL-10.

## Discussion

We recently reported that a single intrathecal injection of MSCs in a transgenic animal model of familial ALS (hSOD1^G93A^) preserved motor functions, extended survival and decreased neuroinflammation at the cellular level [11]. Indeed, the results showed that MSCs integrated into the spinal cord tissue and that microgliosis could be reduced, whereas astrogliosis was not modified. In the present study we confirm that MSC-derived soluble factors inhibit the LPS-mediated induction of the inflammatory markers IL-1β and TNFα in primary microglial cultures. For the *in vivo* experiments, we used an animal model of neuropathic pain caused by PSNL to examine the therapeutic potential of repeated intrathecally injected MSCs. Grafted cells were mostly located in the pia mater spinalis, the meninx surrounding the spinal cord, at day 6 or 21 after injection, while an effect on pain behaviour, microgliosis or cytokine production could not be observed.

Animals undergoing PSNL developed an extended microgliosis in the ipsilateral dorsal horn of the spinal cord and typical behavioural signs of enhanced evoked pain, indicating that the surgery could effectively model neuropathic pain mechanisms. Stem cell-based therapy has been shown to ameliorate pain sensations and also to diminish gliosis in different models of neuropathic pain, including diabetic neuropathy [30], brain injury [31], spinal cord injury (SCI) [21] and PNI such as chronic constriction injury or single ligature nerve constriction [22,32,33]. While after SCI, intraspinal application of neural stem cells could lead to increased allodynia due to aberrant axonal sprouting [34,35], for MSCs this has so far not been reported. Focussing on neuropathic pain after PNI, publications dealing with MSC treatments exclusively describe beneficial outcomes [22,23,36]. While encouraging, these studies are not properly comparable with the present work or with each other as they differ in several aspects of the experimental set-up, such as the number of cells injected, the site and method of injection, the time point of administration (before or after the injury) and the type of injury. Besides diminishing pain behaviour, MSC treatments in PNI also decreased astro- and microglial cell activation as well as reduced levels of pro-inflammatory cytokines, such as IL-1β and IL-17. Furthermore, increased levels of anti-inflammatory cytokines and markers, such as IL-10 and CD206, could be detected [23,36]. The upregulation of CD206 is particularly interesting, as it may witness the phenotypic change of microglia from the pro-inflammatory type M1 to the anti-inflammatory type M2 [37], even though a single marker cannot unequivocally identify the functional orientation of a macrophage or microglial cell [38]. MSCs have already been shown to support this phenotypic switch [39], revealing one of the putative mechanisms of how MSCs exert their anti-inflammatory action. On the basis of these data, it was rather surprising not to detect any significant impact of MSCs on pain behaviour or the associated neuroinflammatory processes in the present study. The lack of effect of MSC treatment in our hands raises questions in regard to the methodical design, where different issues should be reconsidered and discussed, namely: (1) the amount of grafted cells, (2) the timing (time point of treatment/duration of treatment/observation time), (3) the route of administration and (4) the model of inflammation.

While the amount of stem cells used in neuropathic pain studies varies strongly and ranges from 5 × 10^4^ to 10^7^ MSCs [33], we grafted, consistent with our experience in intrathecal injections of cells [11], 10^6^ cells each day but on 3 consecutive days to ensure a constant delivery at a stage where maximal inflammation was expected [29]. It was previously shown that single intraventricular injections of 5 × 10^4^ MSCs resulted in diminished pain-like behaviour and decreased densities of activated astro- and microglial cells [23]. A study testing different cell quantities ranging from 10^4^ to 10^6^ in an ALS mouse model showed that, in general, higher cell numbers are associated with a greater benefit [40]. A similar finding was recently published in a model of experimental neuropathy, but with stem cells from neural origin [32]. Nevertheless, this concept shows limitations as, beyond a certain number of grafted cells, the obtained benefit cannot be augmented by additional injections [41]. Furthermore, as we observed in preliminary experiments, it has been described that high amounts of cells cause clustering in the microinjection cannula, which in turn may cause cell damage and decreased viability [23]. As clustering can be avoided, delivery via catheter injection does not affect the viability or cell function of MSCs [42]. Also, since we decided to deliver the cells locally and not systemically, we are confident that the amount of 10^6^ cells per injection was an appropriate option.

The choice of an adequate time point for cell injection likely constitutes one of the most important issues in the experimental design of stem cell grafting. In the present study we employed repetitive injection of MSCs on days 2, 3 and 4 following PSNL surgery, when a maximal inflammatory response is expected, while other investigators chose single injections on day 4 or 7 after injury [21,23,36]. In the publications of Musolino and colleagues [22] and Klass and colleagues [33], the administration of MSCs coincided with the injury, even though one could argue that this paradigm is hardly transferable to clinical settings. In the latter study, MSCs did not prevent pain, but promoted recovery from pain starting 10 days after injury, leading to a complete reversal, which is contrary to our observations. In this regard, a longer observation period after the treatment could in general be worth considering. However, as the severity of pain is associated with the degree of microglial activation [2], and since we observed a trend for microgliosis attenuation by MSC-treatment 6 days post-PSNL, which was absent again 21 days post-PSNL, a delayed improvement was hardly expected in the present study. Furthermore, we were not able to detect changes in cytokine release caused by PSNL on postoperative day 21, after completion of the behavioural testing of the same animal cohort. This period was presumably too long to detect changes, as we also failed in an earlier study to identify differences in the gene expression of pro-inflammatory genes such as IL-1β, TLR4 and Nox2, 21 days after PSNL [2]. Previous studies describing successful detection of changes in cytokine concentrations using ELISA indeed focussed on earlier time points after nerve ligation or transection, such as 3, 7 or maximally 11 days post-surgery [9,43-45].

Besides the amount of grafted cells, the route of cell administration also prevalently differs in the literature and should be a main topic while discussing the study design. Frequently, cell delivery in rats is carried out by systemic injection via the tail vein, which represents a less invasive administration route. When choosing this approach, the amount of grafted cells usually should be rather high as the majority will be trapped in the lungs or be cleared by peripheral macrophages before reaching their destination [46]. Surprisingly, studies using intravenous injection of MSCs in neuropathic pain reported striking results, such as complete reversal of hyperalgesia [33]. Furthermore, MSCs were found to accumulate in the L4-L5 spinal cord and pre-frontal cortex, revealing their specificity for engraftment in neuropathic pain controlling and processing areas [36]. In regard to the use in neuropathic pain models, local administration of MSCs by intraganglionic [22], intraparenchymal [21] or intraventricular [23] injections have been reported, whereas intrathecal approaches have not been tested so far. While one could speculate that direct delivery of cells into the cerebrospinal fluid at the L4-L5 level of the spinal cord would improve the migration to the site of inflammation as well as the integration into the tissue, we had to realise that tissue infiltration was here far less efficient compared to other ways of administration. Interestingly, the same observation has been made for the intrathecal delivery of MSCs after SCI, when cells were injected within the acute inflammatory phase (days 1 to 7) [47]. Here, grafted cells were also found attached to the pia mater or accumulated around the anterior spinal artery. In contrast, rats injected repeatedly within the subacute phase (days 7 to 14) on days 7, 8 and 9 after SCI showed functional recovery, while MSCs incorporated into the central lesion. One of our initial hypotheses proposed that MSCs would be attracted by mediators released during inflammation, promoting their migration to the site of inflammation, in our case to the ipsilateral dorsal horn. While MSCs were detected at the lumbar level (L4-L5) of the spinal cord, these rarely entered the tissue, but were “trapped” in the pia mater spinalis, suggesting that the inflammation caused by PSNL might not have been strong or long enough to attract the cells. Indeed, it has been demonstrated that the degree of injury is important for the survival and tissue integration of MSCs [21,48]. This might also explain why our results differ from our former study in ALS. While PSNL causes a rather acute inflammation, ALS-associated neuroinflammation is a chronic process, which accompanies the disease onset and increases continuously until end-stage. Thus, we can only hypothesise that important features of chronic inflammation, which might be essential for the proper attraction of MSCs from the cerebrospinal fluid and their migration into the nervous parenchyma were still absent in the model of peripheral nerve ligature.

Taken together, we may only suggest that MSC penetration through the pia mater spinalis and infiltration into the inflamed spinal cord parenchyma might be more challenging than expected. Cizkova and colleagues [47] speculated that early injected MSCs cannot integrate into the spinal cord parenchyma as they are repelled by a too strong inflammatory response after injury. Nevertheless, we rather hypothesise that the mechanical border surrounding the spinal cord surface, comprising the pia mater spinalis, its basal membrane and the subjacent glia limitans superficialis, is preserved intact during the first days of inflammation. This idea is supported by the fact that within the first 7 days after SCI, due to inflammatory processes, the basement membrane is drastically altered and astrocytic foot processes, which compose the glia limitans, retract from the basement membrane. The progression of these events even coincides with a pronounced infiltration of inflammatory cells into the spinal cord parenchyma [49,50].

## Conclusion

Repetitive intrathecal delivery of MSCs on day 2, 3 and 4 after PSNL neither affected pain sensations such as allodynia and hyperalgesia, nor decreased microglial activation that developed in the ipsilateral spinal cord. Twenty-one days after grafting, labelled MSCs could be relocated in the pia mater spinalis, but did not integrate into the spinal cord parenchyma. Thus, early intrathecal delivery of MSCs in the specific model of PSNL has no beneficial effects on neuropathic pain or the underlying neuroinflammatory processes. After critical analysis of our data and confrontation with the literature, we are convinced that the amount of cells and the time points of administration were chosen correctly in the present study. Nevertheless, the selected application route of intrathecal injection seems inefficient in regard to the intensity level of neuroinflammation in the spinal cord after PSNL. To accurately evaluate the therapeutic potential of MSCs in this specific model, further studies should focus on the reassessment of alternative administration routes.

## Abbreviations

ALS: amyotrophic lateral sclerosis
BrdU: bromodeoxyuridine
DMEM: Dulbecco’s modified Eagle’s medium
FBS: foetal bovine serum
HS: horse serum
Iba1: ionized calcium-binding adapter molecule 1
IL: interleukin
LPS: lipopolysaccharide
MSC: mesenchymal stem (stromal) cells
PBS: phosphate-buffered saline
PCR: polymerase chain reaction
PNI: peripheral nerve injury
PSNL: partial sciatic nerve ligation
PWL: paw withdrawal latencies
PWT: paw withdrawal threshold
RT: room temperature
SCI: spinal cord injury
TLR: Toll-like receptor
TNF: tumour necrosis factor
